# Significant association between admission serum monocyte chemoattractant protein-1 and early changes in myocardial function in patients with first ST-segment elevation myocardial infarction after primary percutaneous coronary intervention

**DOI:** 10.1186/s12872-019-1098-z

**Published:** 2019-05-10

**Authors:** Yong Zhu, Chengping Hu, Yu Du, Jianwei Zhang, Jinxing Liu, Hongya Han, Yingxin Zhao

**Affiliations:** Department of cardiology, Beijing Anzhen Hospital, Capital Medical University, #2, Anzhenlu, Chaoyang District, Beijing, 100029 China

**Keywords:** STEMI, MCP-1, LVEF, Hs-cTnI, I/R injury

## Abstract

**Background:**

Recent studies have indicated that monocyte chemoattractant protein-1 (MCP-1) plays an important role in the initiation and progression of ischaemic heart disease. However, no previous research has investigated the correlation between serum MCP-1 levels and early changes in myocardial function in patients with ST-segmental elevation myocardial infarction (STEMI) after primary percutaneous coronary intervention (PCI).

**Methods:**

A total of 87 STEMI patients who had undergone a successful primary PCI were consecutively recruited. All the patients included in this study were grouped into two subgroups according to the median value of MCP-1 upon admission. An early change in left ventricular ejection fraction (LVEF) was defined as (LVEF at 3 months post-STEMI)-(LVEF at 2 days post-STEMI).

**Results:**

Serum MCP-1 levels increased gradually over time during the first 72 h after the onset of STEMI. The concentration of hypersensitive cardiac troponin I (hs-cTnI) upon admission as well as at 24 h and 72 h after primary PCI, especially the peak hs-cTnI concentration, declined significantly in the low admission MCP-1 group. As continuous variable, admission MCP-1 also correlated positively with admission hs-cTnI, hs-cTnI at 24 h after primary PCI, and peak hs-cTnI. Additionally, the absolute early change in LVEF improved markedly in the low admission MCP-1 group (3.77% ± 6.05% vs − 0.18% ± 7.69%, *p* = 0.009) compared to that in the high admission MCP-1 group. Most importantly, the global LVEF in the low admission MCP-1 group also improved significantly at 3 months compared to baseline LVEF (55.79% ± 7.05% vs 59.60% ± 6.51%, *p* = 0.011), while an improvement in global LVEF was not observed in the high admission MCP-1 group. Furthermore, as a continuous variable, the MCP-1 level up admission also correlated negatively with early changes in LVEF (*r* = − 0.391, *p* = 0.001). After assessment by multiple linear regression analysis, the MCP-1 level upon admission remained correlated with early changes in LVEF [beta = − 0.089, 95% CI (− 0.163 to − 0.015), *p* = 0.020].

**Conclusion:**

MCP-1 upon admission not only correlated positively with hs-cTnI at different time points and peak hs-cTnI, but also associated inversely with early improvements in myocardial function in patients with first STEMI. So we speculated that suppression the expression of MCP-1 via various ways may be a promising therapeutic target in myocardial I/R injury in the future.

## Background

Recently, multiple clinical and experimental evidence have supported inflammation as a critical regulatory process that links multiple traditional risk factors such as hypertension, obesity and insulin resistance with the initiation and progression of coronary artery disease (CAD) [1, 2]. The inflammatory response is more complicated in regard to ST-element elevation myocardial infarction (STEMI), which involves the coordinated activation of a series of inflammatory cytokines and has a profound influence on myocardial ischaemia/reperfusion (I/R) injury, leading to subsequent negative remodelling [3, 4]. Therefore, it is necessary to elucidate the association between inflammatory cytokines and adaptive/maladaptive myocardial healing processes and to determine their benefits in clinical practice.

Chemokines are a subset of inflammatory cytokines that participate in the recruitment of leukocytes to sites of inflammation and are grouped into subfamilies: CXC chemokines and CC chemokines [5]. As one of the most well-studied CC chemokines, monocyte chemoattractant protein-1(MCP-1) has shown a significant association with risk factors for atherosclerosis such as age, hypertension, diabetes and renal insufficiency [6–8]. In addition, MCP-1 may initiate the formation and progression of atherosclerosis by directly stimulating monocyte infiltration and the accumulation of lipid-laden foam cells [5, 9]. Most importantly, recent evidence from animal models indicated that MCP-1 may play an important role in myocardial I/R injury and negative remodelling through diverse potential mechanisms [10, 11]. However, no previous research has addressed this topic in humans. Therefore, in this research, we investigated the serial change in serum MCP-1 in patients with STEMI and determined the association between the time course of serum MCP-1 and early changes in myocardial function.

## Method

### Subjects

The present cross-sectional research recruited 87 patients with STEMI from two major hospitals in Beijing, China (Beijing Anzhen Hospital and Beijing Daxing Hospital, Capital Medical University) between August 2017 and September 2018. The patients recruited all met the following inclusion criteria: 18 years < age < 80 years, typical chest pain lasting for more than 20 min and no more than 12 h, electrocardiography indicating ST-segment elevation in at least two contiguous leads and primary percutaneous coronary intervention (PCI) performed successfully by experienced interventionists. We excluded patients with any of the following criteria: severe acute heart failure (Killip class III and IV), prior history of myocardial infarction, previous history of coronary artery bypass graft and PCI, rescue angioplasty, current acute infection, malignancy, chronic inflammatory disease and autoimmune disease. Lastly, all the patients recruited were grouped into two subgroups according to the median value of admission MCP-1 level at admission (47.85 pg/ml). Patients with MCP-1 admission levels < 47.85 pg/ml were regarded as the low admission MCP-1 group, while patients with MCP-1 admission levels ≥47.85 pg/ml were considered the high admission MCP-1 group.

Before initiation of the present study, we obtained approval from the ethics committee of Beijing Anzhen Hospital, Beijing Da Xing Hospital, Capital Medical University. In addition, we also obtained informed written consent from the recruited patients.

### Primary PCI and medication

Before the primary PCI, all the patients included in the present study were pre-treated with a leading dose of aspirin (300 mg) and clopidogrel (600 mg). In addition to antiplatelet therapy, all the patients recruited also received weight-adjust intravenous heparin (70–100 U/kg). The primary PCI was performed according to the newest guidelines. Thrombus aspiration, as well as the choice of a new generation drug-eluting stent (DES) or a glycoprotein IIb/IIIa inhibitor, were left to operator discretion. After the primary PCI, all the patients received dual antiplatelet therapy (aspirin 100 mg and clopidogrel 75 mg). Additionally, patients were also prescribed with statins, β-blockers, and an angiotensin receptor blocker (ARB) or angiotensin-converting enzyme inhibitor (ACEI).

### Clinical data collection

We obtained the demographic and clinical data including age, sex, height, weight, body mass index (BMI), blood pressure and Killip class upon admission as well as medical history and medication use through hospital records. In addition to total ischaemia time, angiography and procedural data including single or multiple vessel disease, the number of DES and thrombolysis in myocardial infarction (TIMI) flow grade were also obtained. BMI was measured as the ratio of weight (kilogram) to height squared (meter). Total ischaemia time was defined as the time from symptom onset to recanalization of the infarction-associated artery. In addition to the infarction-associated artery, patients with 50% or greater stenoses in other major epicardial vessels were considered to have multiple vessel diseases.

### Blood sample measurement

Venous blood samples were obtained upon admission as well as at 24 h and 72 h after primary PCI, followed by centrifugation for 15 min at 2500 rpm. Then, the serum samples were stored at − 80 °C until analysis. Routine blood, urine, and biochemical parameters were all measured in the central laboratory of the Beijing Anzhen Hospital.

In our present study, the concentration of serum MCP-1 and hypersensitive cardiac troponin I (hs-cTnI) were measured in duplicate by commercially available enzyme-linked immunosorbent assay (ELISA) kits (Blue Gene, Shanghai, China). The intra-assay and inter-assay coefficients of variation were both < 5%.

### Echocardiography

All the patients recruited underwent echocardiography examination by experienced physicians and technicians who were blinded to the clinical data of all patients at 2 days and 3 months post-STEMI. With the supervision of experienced cardiologists, we obtained a standard echocardiography view. Subsequently, we obtained the left ventricular ejection fraction (LVEF) via the modified biplane Simpson method.

### Statistical analysis

Normally distributed continuous variables were expressed as the mean ± SD, and skewed continuous variables are shown as the median [interquartile range (IQR)]. The difference between normally distributed continuous variables was tested by an unpaired t-test, while skewed variables were compared by the Mann-Whitney U test. Categorical variables are expressed as numbers (%) and were compared by a Chi square test. The association between variables was tested by Spearman correlation testing. Univariate analysis and multivariate linear regression analysis were used to assess the association between the change in LVEF from baseline to 3 months post-STEMI and other variables. All the statistical analyses were performed with SPSS 20.0 software (SPSS Inc., Chicago, IL, USA). *P* < 0.05 was regarded as statistically significant.

## Results

### Baseline characteristics

The baseline characteristics are presented in Table 1. The mean age was 55.51 ± 11.76 years, and 74.7% of the patients were male. For atherosclerosis risk factors, the prevalence of current smoking, hypertension, and diabetes was 69.0, 53.6 and 18.4%, respectively. Most importantly, the total ischaemia time was 278.00 (197.00, 412.00) minutes, which indicated that all the patients recruited underwent primary PCI timely. For the culprit lesion, 56.9% involved the left anterior descending coronary artery (LAD). Additionally, 50.6% of the patients recruited had multiple vessel disease, and an initial TIMI flow grade of 0 was observed in 86.2% of the patients included. Finally, 98.9% of patients were treated with new generation DES, and the mean number of stents was 1.09 ± 0.33.

**Table 1:** Baseline Characteristics

Variables	Total	Low MCP-1 Group	High MCP-1 Group	*p*-value
Number	87	43	44	
Age (years)	55.51 ± 11.76	55.26 ± 11.42	55.75 ± 12.21	0.846
Male (%)	65 (74.7%)	35 (81.4%)	30 (68.2%)	0.218
BMI (Kg/m^2^)	26.07 ± 3.35	25.93 ± 3.54	26.20 ± 3.19	0.709
Systolic BP (mmHg)	115.83 ± 18.17	117.56 ± 18.03	114.14 ± 18.35	0.383
Diastolic BP (mmHg)	74.03 ± 13.44	75.26 ± 13.66	72.84 ± 13.28	0.405
Total ischemia time (minute)	278.00 (197.00,412.00)	376.53 ± 207.58	319.50 ± 185.33	0.180
Killip class II on admission (%)	39 (44.8%)	22 (51.2%)	17 (38.6%)	0.284
LVEF at 2 days post-STEMI (%)	59.00 (53.00,63.00)	55.79 ± 7.05	59.16 ± 5.64	0.016
Current Smoker (%)	60 (69.0%)	32 (74.4%)	28 (63.6%)	0.355
Hypertension (%)	44 (53.6%)	20 (46.5%)	24 (54.5%)	0.523
Diabetes (%)	16 (18.4%)	3 (7.0%)	13 (29.5%)	0.011
Laboratory examination				
White blood cell (10^9^/L)	11.21 ± 3.07	11.47 ± 3.23	10.96 ± 2.93	0.445
Hemoglobin (g/L)	139.17 ± 18.77	141.93 ± 16.49	136.48 ± 20.60	0.177
Platelet (10^9^/L)	235.78 ± 57.78	233.63 ± 51.84	237.89 ± 63.58	0.733
FBG (mmol/L)	6.40 (5.40,7.85)	6.10 (5.10,7.40)	6.40 (5.63,9.95)	0.244
Triglycerides (mmol/L)	1.45 (1.01,2.06)	1.23 (0.91,1.63)	1.59 (1.16,2.37)	0.036
LDL-C (mmol/L)	2.79 ± 0.76	2.72 ± 0.75	2.86 ± 0.78	0.404
HDL-C (mmol/L)	0.99 ± 0.21	0.98 ± 0.21	1.00 ± 0.20	0.647
Peak NT-proBNP (pg/ml)	641.00 (322.00,1504.00)	641.00 (384.00,1554.00)	657.50 (253.25,1433.75)	0.610
Angiography data				
Culprit				0.017
LAD (%)	49 (56.3%)	30 (69.8%)	19 (43.2%)	
Others(%)	38 (43.7%)	13 (30.2%)	25 (56.8%)	
Number of diseased vessels				0.376
1- vessel disease(%)	43 (49.4%)	24 (55.8%)	19 (43.2%)	
2- vessel disease(%)	26 (29.9%)	10 (23.3%)	16 (36.4%)	
3- vessel disease(%)	18 (20.7%)	9 (20.9%)	9 (20.5%)	
TIMI = 0, before procedure	75 (86.2%)	37 (86.0%)	38 (86.4%)	*p* > 0.99
Procedure data				
Stent implantation(%)	86 (98.9%)	43 (100.0%)	43 (97.7%)	*p* > 0.99
The number of stent	1.09 ± 0.33	1.12 ± 0.32	1.07 ± 0.33	0.498
Thrombus aspiration(%)	10 (11.5%)	5 (11.6%)	5 (11.4%)	*p* > 0.99
Tirofiban(%)	57 (65.5%)	26 (60.5%)	31 (70.5%)	0.372
TIMI = 3 (After procedure)	66 (75.9%)	36 (83.7%)	30 (68.2%)	0.132
Medication				
Aspirin(%)	87 (100.0%)	43 (100.0%)	44 (100.0%)	*p* > 0.99
Clopidogrel(%)	87 (100.0%)	43 (100.0%)	44 (100.0%)	*P* > 0.99
Stains(%)	87 (100.0%)	43 (100.0%)	44 (100.0%)	*p* > 0.99
β-block(%)	77 (88.5%)	37 (86.0%)	40 (90.9%)	0.521
ACEI/ARB(%)	60 (69.0%)	29 (67.4%)	31 (70.5%)	0.819

Data are presented as Mean ± SD, median (low quartile,upper quartile),or number (%)

MCP-1 monocyte chemoattractant-1, BMI body mass index, BP blood pressure, LVEF left ventricular ejection fraction, FBG fasting blood glucose, LDL-C low-density lipoprotein cholesterol, HDL-C high-density lipoprotein cholesterol, NT-proBNP N-terminal pro B-type natriuretic peptide, LAD left anterior descending coronary artery, TIMI thrombolysis in myocardial infarction, ACEI/ARB angiotensin-converting enzyme inhibitor/angiotensin receptor blocker

Additionally, the patients recruited were grouped into two subgroups according to the median value of MCP-1 level upon admission. We observed that the low admission MCP-1 group had a lower baseline LVEF (55.79 ± 7.05% vs 59.16% ± 5.64%, *p* = 0.016) and a lower prevalence of diabetes (7.0% vs 29.5%, *p* = 0.011) compared to the high admission MCP-1 group. The low admission MCP-1 group had a high prevalence of culprit vessel lesions in the LAD (69.8% vs 43.2%, *p* = 0.017), which may account for the low baseline LVEF.

### Change in serum MCP-1 levels in patients with STEMI

**Fig. 1:**
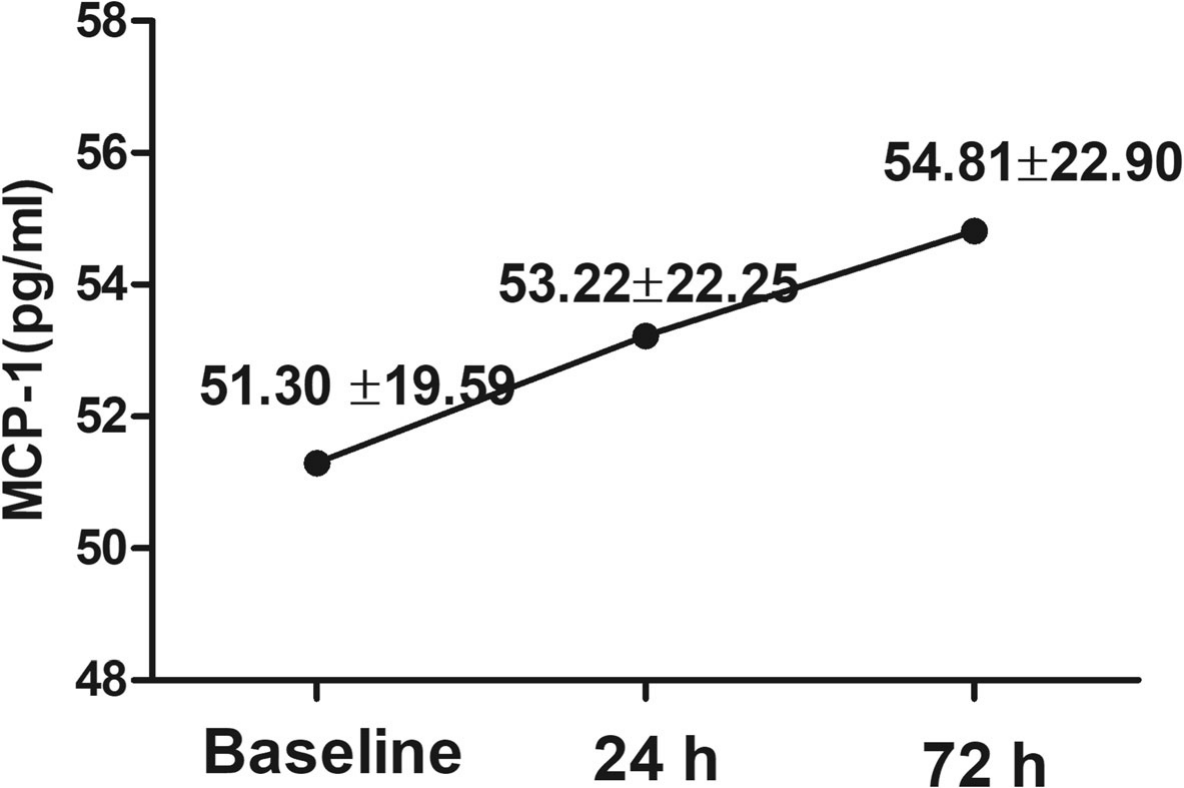
The time course of serum MCP-1 concentration during the first 72 h in patients with STEMI. STEMI, ST-segment elevation myocardial infarction; MCP-1, monocyte chemoattractant protein-1

The mean MCP-1 concentration in patients with STEMI upon admission as well as at 24 h and 72 h after primary PCI was 51.30 ± 19.59 pg/ml, 53.22 ± 22.25 pg/ml and 54.81 ± 22.90 pg/ml, respectively. As shown in Fig. 1, a sustained increase in serum MCP-1 concentration was observed in patients with STEMI. Additionally, a positive correlation was observed between the MCP-1 level at admission and blood glucose (*r* = 0. 331, *p* = 0.002) and triglycerides (*r* = 0.222, *p* = 0.039) upon admission, while neither the MCP-1(24 h) nor MCP-1(72 h) level had any association with blood glucose and triglycerides upon admission. Furthermore, the MCP-1 levels at all time points had no significant associations with the total ischaemia time.

### Low admission MCP-1 level limits myocardial injury

**Fig. 2:**
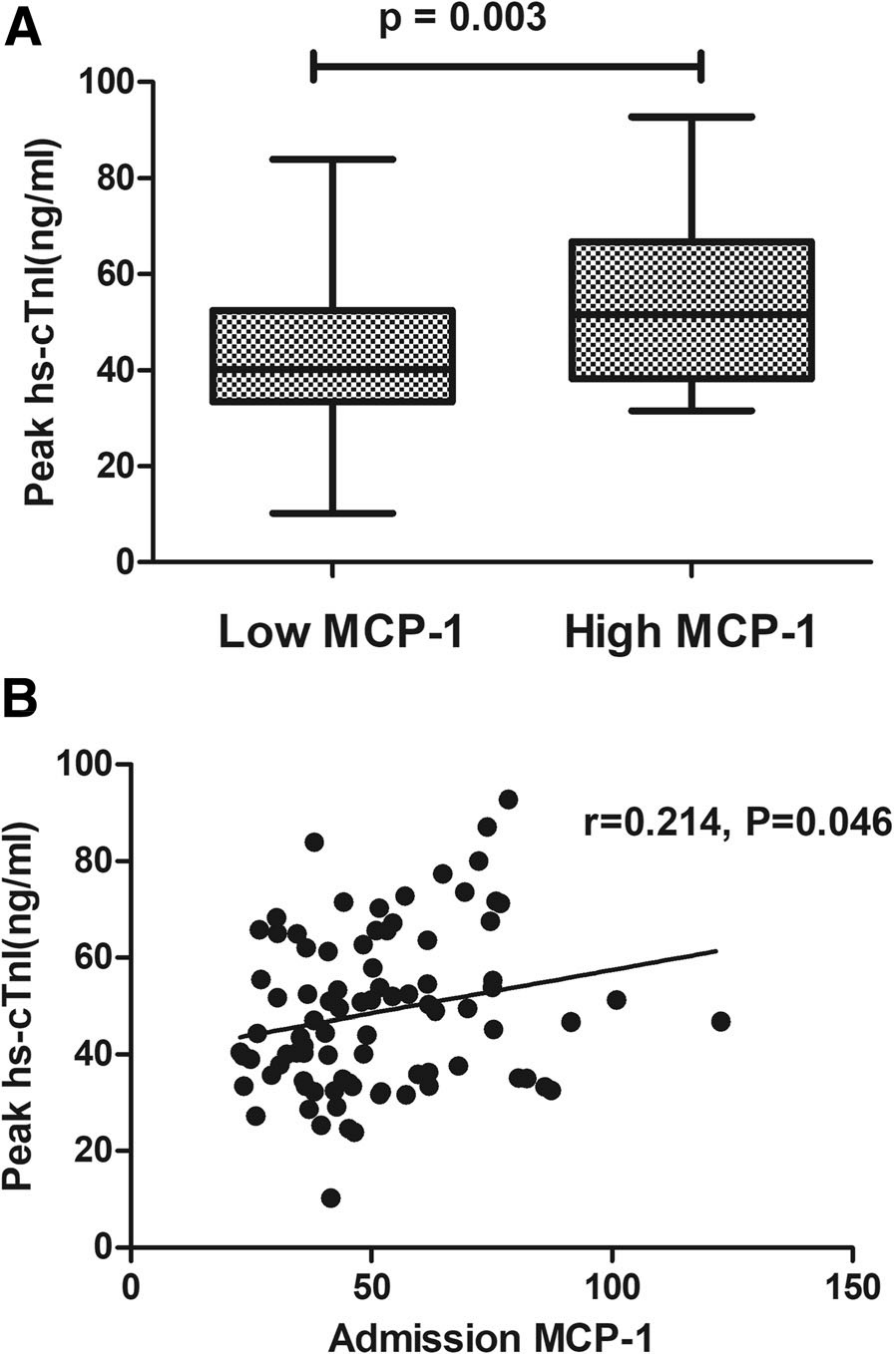
The peak hs-cTnI concentration in the low admission MCP-1 and high admission MCP-1 groups (**a**). The correlation between the MCP-1 level upon admission and the peak hs-cTnI level (**b**). hs-cTnI, hypersensitive cardiac troponin I; MCP-1, monocyte chemoattractant protein-1

The hs-cTnI concentration upon admission (36.17 ± 12.70 pg/ml vs 45.36 ± 14.39 pg/ml, *p* = 0.002), as well as at 24 h (31.90 ± 14.67 pg/ml vs 42.27 ± 15.55 pg/ml, *p* = 0.002) and 72 h (38.60 ± 14.75 pg/ml vs 48.17 ± 16.28 pg/ml, *p* = 0.005) after primary PCI were all significantly lower in the low admission MCP-1 group than in the high admission MCP-1 group. Additionally, as shown in Fig. 2 A, the peak hs-cTnI was also lower in the low admission MCP-1 group (43.45 ± 15.02 pg/ml vs 53.84 ± 16.16 pg/ml, *p* = 0.003). We also found that the MCP-1 level upon admission had a positive correlation with the hs-cTnI level upon admission (*r* = 0.268, *p* = 0.012), as well as at 24 h (*r* = 0.253, *p* = 0.018) after primary PCI, and it had a trend with hs-cTnI at 72 h (*r* = 0.155, *p* = 0.151) after primary PCI. Most importantly, as shown in Fig. 2 B, the MCP-1 level upon admission was also positively correlated with peak hs-cTnI (*r* = 0.214, *p* = 0.046).

Additionally, we divided all the patients into two subgroups according to the median value of MCP-1 (24 h). The hs-cTnI level at 72 h after the procedure (39.34 ± 14.75 pg/ml vs 47.45 ± 16.67 pg/ml, *p* = 0.018) declined markedly in the low MCP-1 group (24 h). The hs-cTnI level upon admission and at 24 h after the procedure as well as the peak hs-cTnI level were all decreased in the low MCP-1 group (24 h), although the data were not significantly different. For MCP-1 (72 h), we also found that the low MCP-1 group had a low hs-cTnI level upon admission as well as at 24 h and 72 h after primary PCI, and the peak hs-cTnI level was low, even though the difference was not significant.

### MCP-1 and the change in LVEF during follow-up

**Fig. 3:**
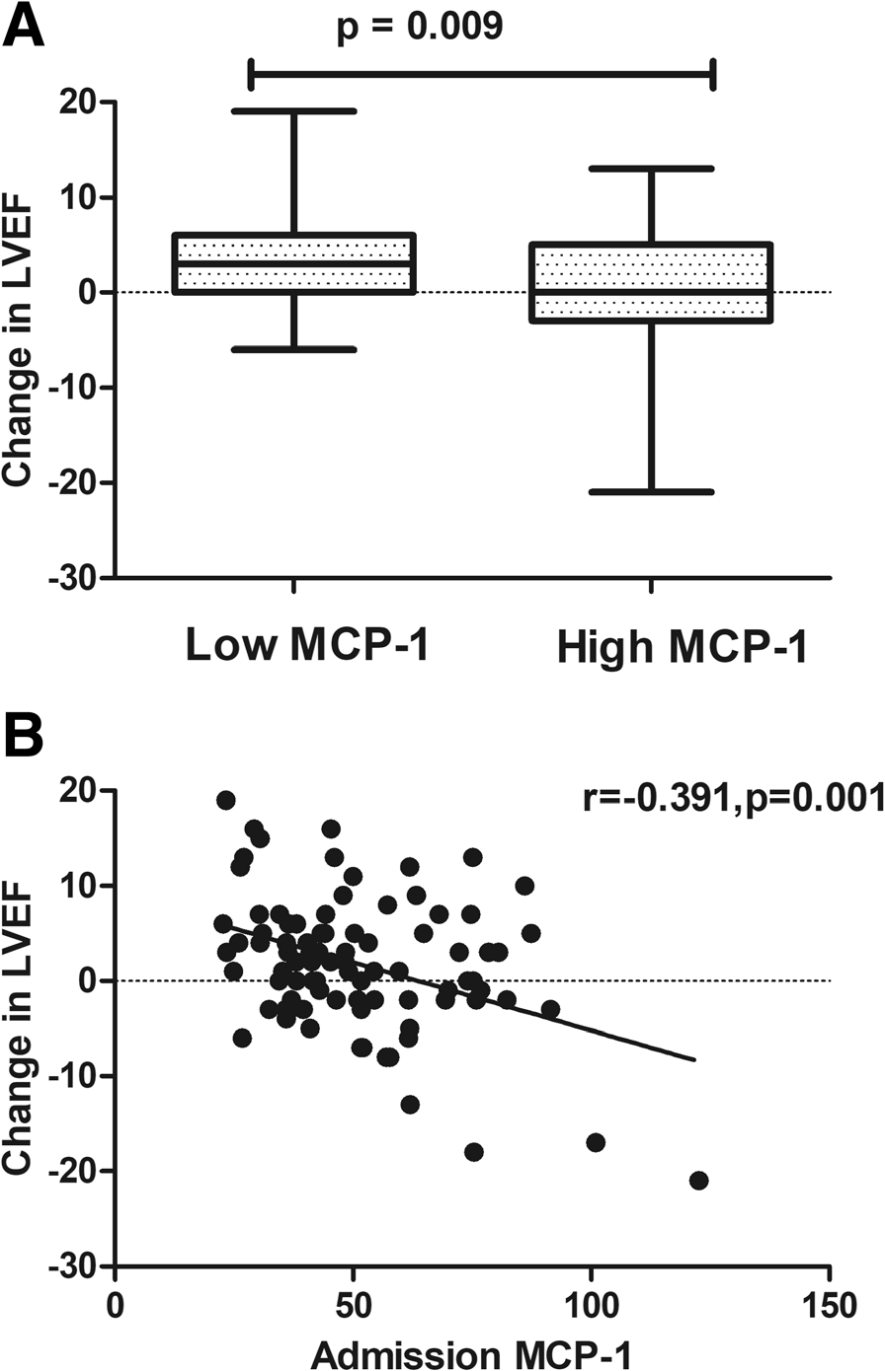
Early changes in LVEF in the low admission MCP-1 group and high admission MCP-1 group (**a**). The association between the MCP-1 level upon admission and early changes in LVEF (**b**). LVEF, left ventricular ejection fraction; MCP-1, monocyte chemoattractant protein-1

**Fig. 4:**
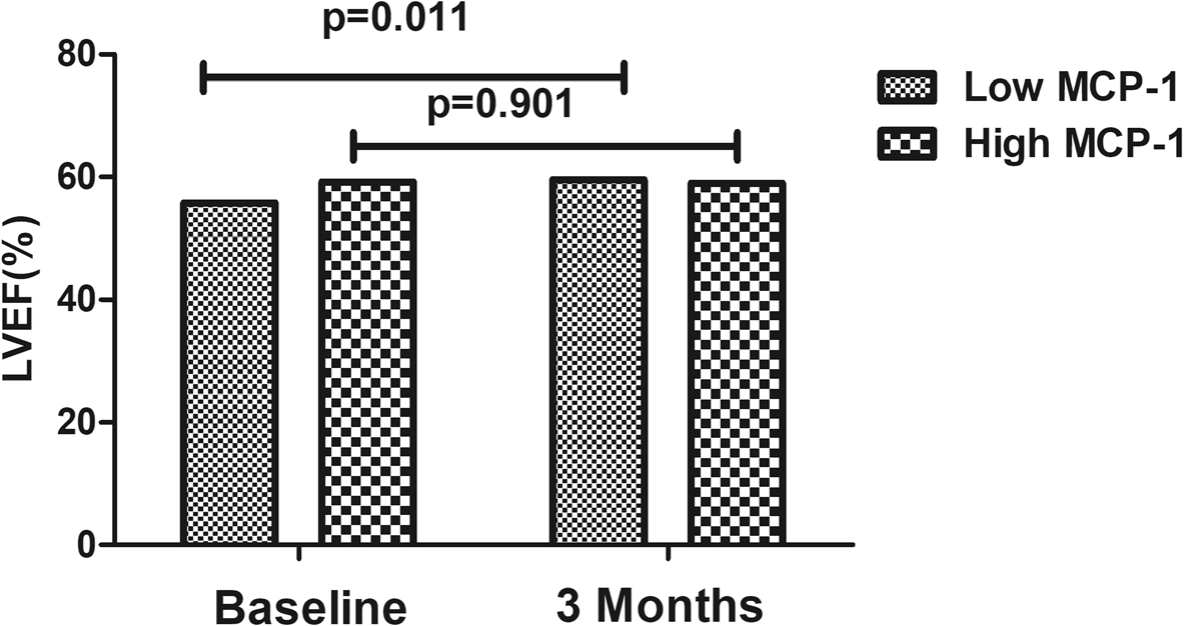
The LVEF in patients in the low and high MCP-1 groups at baseline and 3 months post-STEMI. LVEF, left ventricular ejection fraction; MCP-1, monocyte chemoattractant protein-1; STEMI, ST-segment elevation myocardial infarction

The global baseline LVEF (55.79% ± 7.05% vs 59.16% ± 5.64%, *p* = 0.016) was low in the low admission MCP-1 group due to the high prevalence of culprit vessel lesions in LAD, at least in part (69.8% vs 43.12%, *p* = 0.017). However, as shown in Fig. 3a, the absolute change in LVEF from baseline to 3 months post-STEMI improved significantly in the low admission MCP-1 group (3.77 ± 6.05% vs − 0.18 ± 7.69%, *p* = 0.009). Furthermore, as shown in Fig. 3b, the MCP-1 level upon admission was also negatively correlated with early changes in LVEF from baseline to 3 months post-STEMI (*r* = − 0.391, *p* = 0.001). Additionally, as shown in Fig. 4, the global LVEF in the low admission MCP-1 group also improved markedly (55.79 ± 7.05% vs 59.60 ± 6.51%, *p* = 0.011), while the high admission MCP-1 group did not present such an improvement (59.16 ± 5.64% vs 58.98% ± 7.83%, *p* = 0.901).

For MCP-1 (24 h), the low MCP-1 group also exhibited a large change in LVEF (3.51% ± 6.59% vs 0.07% ± 7.37, *p* = 0.024) and a significant improvement in LVEF (55.42% ± 7.20% vs 58.98 ± 7.17%, *p* = 0.024) at 3 months post-STEMI. Additionally, MCP-1 (24 h) was also negatively correlated with early changes in LVEF (*r* = − 0.282, *p* = 0.008), while MCP-1(72 h) had no significant association with the change in LVEF.

### Univariate analysis and multivariate linear regression analysis

To determine the association among the MCP-1 level upon admission, MCP-1 (24 h) and early changes in LVEF, we performed univariate analysis and multivariate linear regression analysis. As shown in Table 2, where the data were adjusted for other confounding factors including age, sex, diabetes and baseline LVEF, the MCP-1 level upon admission was associated with early changes in LVEF [beta = − 0.089, 95% CI (− 0.163 to − 0.015), *p* = 0.020], while the MCP-1 level (24 h) was not [beta = − 0.039, 95% CI (− 0.016 to 0.027), *p* = 0.245].

**Table 2:** Correlation between the global change in LVEF and other variables using univariate analysis and multivariate linear regression analysis

Variables	The global change in LVEF from baseline to 3 months					
Model A		Univariate		Multivariate		
				(R^2^ = 0.309)		
	Beta	95% CI	p	Beta	95% CI	p
Age	−0.023	−0.154 to 0.108	0.729	− 0.048	− 0.165 to 0.068	0.410
Sex	−3.160	−6.630 to 0.310	0.074	−1.823	−5.151 to 1.505	0.279
Diabetes	−4.007	−7.879 to −0.135	0.043	−0.184	− 3.947 to 3.580	0.923
Baseline LVEF	−0.501	−0.711 to − 0.292	0.001	−0.447	− 0.664 to − 0.230	0.001
Baseline MCP-1	− 0.143	−0.216 to − 0.070	0.001	−0.089	− 0.163 to − 0.015	0.020
TIMI flow (After procedure)	0.820	−0.365 to 2.004	0.172			
Model B		Univariate		Multivariate		
				(R^2^ = 0.273)		
	Beta	95% CI	p	Beta	95% CI	p
Age	−0.023	−0.154 to 0.108	0.729	−0.043	− 0.165 to 0.078	0.482
Sex	−3.160	−6.630 to 0.310	0.074	−2.463	−5.817 to 0.890	0.148
Diabetes	−4.007	−7.879 to −0.135	0.043	−0.558	−4.424 to 3.308	0.775
Baseline LVEF	−0.501	−0.711 to − 0.292	0.001	−0.478	− 0.701 to − 0.255	0.001
MCP-1 (24 h)	− 0.091	−0.158 to − 0.024	0.008	−0.039	− 0.106 to 0.027	0.245
TIMI flow (After procedure)	0.820	−0.365 to 2.004	0.172			

LVEF left ventricular ejection fraction, MCP-1 monocyte chemoattractant protein-1, TIMI thrombolysis in myocardial infarction, CI confidence interval

Age, sex and other variables with p < 0.05 in univariate analysis were included in multivariate linear regression model

## Discussion

To our knowledge, this is the first study that evaluates the correlation between the time course of MCP-1 levels and early changes in myocardial function in patients with STEMI. First, a sustained elevation in the MCP-1 serum level was observed in the patients recruited during the first 72 h following primary PCI. Second, we show that the MCP-1 level upon admission was predictive of a later change in necrotic markers, while MCP-1 at 24 h and 72 h may not be. Finally, the MCP-1 level upon admission and MCP-1 (24 h) had an inverse correlation with early improvements in myocardial function, but MCP-1 (24 h) was not associated with the early improvement in myocardial function after adjusting for other confounding factors.

Evidence from experimental and clinical studies revealed that C-C chemokines, specifically MCP-1, play crucial pathogenic roles in ischaemic heart disease [12–15]. Recent evidence from John. Parissis et.al showed that serum MCP-1 was continuously elevated in patients with acute myocardial infarction (AMI) during a 7-day hospitalization period compared to that in healthy people [16]. In our present research, serum MCP-1 concentration increased gradually over time in patients with STEMI. Furthermore, Tadashi Kakio et.al pointed out that the amount of MCP-1 mRNA-positive cells in rat hearts increased significantly after reperfusion [17]. This evidence indicated that MCP-1 may play a role in myocardial I/R injury. Additionally, our present research revealed that the MCP-1 level upon admission was positively associated with blood glucose and triglycerides upon admission, while MCP-1 levels at 24 h and 72 h after primary PCI had no such positive correlation with admission blood glucose and triglycerides; this may be partially due to the drastic change in MCP-1 concentration after reperfusion [17], as Tadashi Kakio et.al reported in their research.

Additionally, in our present research, the hs-cTnI concentration at each time point and the peak hs-cTnI level, which were associated with infarction size [18, 19], both decreased significantly in the low admission MCP-1 group. Similar to our finding, Takanori Hayaski et.al showed that genetic knockout of C-C chemokine receptor-2 (CCR-2), which binds solely with MCP-1 in mice, leads to a marked decrease in infarct size compared to wild-type mice after reperfusion [20]. Furthermore, there is evidence indicating that the neutralization of MCP-1 with anti-MCP-1 reduced the infarct size markedly in mice [10, 11]. In addition, there is also previous research suggesting that peak MCP-1 levels increase significantly in patients with acute myocardial infarction (AMI) complicated by severe left ventricular dysfunction compared to other patients with AMI [16]. In vivo and in vitro experiments with the genetic knockout of both MCP-1 and anti-MCP-1 retarded the progression of left ventricular dilation and dysfunction [10, 11]. Our present research further illustrates that early changes in LVEF from baseline to 3 months post-STEMI improved significantly in the low admission MCP-1 group compared to that in the high admission group. Therefore, the evidence above all indicates that MCP-1 may promote myocardial I/R injury and post-infarction remodelling.

The suppression of proinflammatory cytokines synthesis, reduction of macrophage activation and decreased myofibroblast infiltration may account, at least in part, for reducing myocardial I/R injury and retarding left ventricular remodelling in the low admission MCP-1 group [10]. In addition, low MCP-1 levels upon admission may suppress myocardial I/R injury and subsequent negative remodelling through the suppression of macrophage-associated oxidative stress, as Takanori Hayasaki et.al reported [20]. There is evidence indicating that low MCP-1 levels upon admission may suppress I/R-induced myocardial apoptosis [11], which also contributes to myocardial I/R injury, and the progression of negative remodelling. Similar to myocardial apoptosis, autophagy also accounts, at least in part, for I/R injury [21]. Whether MCP-1 has effects on autophagy calls for future studies. In addition, recent research indicated that an imbalance between proinflammatory adipocytokines and anti-inflammatory adipocytokines also contributes to myocardial I/R injury [22–25]. Therefore, it is necessary for future studies to further investigate the influence of MCP-1 on the imbalance between pro- and anti-inflammatory adipocytokines.

The present research has several limitations. First, this is a cross-sectional study with a relatively small sample size that fails to elucidate the causal relationship between MCP-1 levels upon admission and I/R injury. Second, the patients included in this research all meeting the relatively strict inclusion or exclusion criteria to decrease the heterogeneity of the participants, which maybe account for the finding that patients included in our present research were relatively young compared to other STEMI studies and make the results from this research unsuitable for the general population. Third, we did not apply cardiac magnetic resonance imaging or single-photon emission computed tomography imaging to assess LVEF. Finally, the serum receptor CCR-2 that binds solely to MCP-1 was not measured.

## Conclusion

In the present research, we demonstrated for the first time that MCP-1 upon admission had a positive association with hs-cTnI at different time points and peak hs-cTnI and had inverse correlation with early improvements in myocardial function in patients with first STEMI. Therefore, the suppression of MCP-1 synthesis via various mays such as genetic knockout of MCP-1 and neutralization of MCP-1 with anti-MCP-1 may be an effective therapy for I/R injury and negative remodelling post-infarction in the future. While we know little about MCP-1 in patients with STEMI at present, future studies are warranted to elucidate the definitive mechanisms through which MCP-1 exerts physiological effects in the setting of STEMI.

## Abbreviations

AMI: Acute myocardial infarction
ARB or ACEI: Angiotensin receptor blocker or angiotensin-converting enzyme inhibitor
BMI: Body mass index
CAD: Coronary artery disease
CCR-2: C-C chemokine receptor-2
DES: Drug-eluting stent
hs-cTnI: Hypersensitive cardiac troponin I
I/R: Ischaemia and reperfusion
LAD: Left anterior descending coronary artery
LVEF: Left ventricular ejection fraction
MCP-1: Monocyte chemoattractant protein-1
PCI: Percutaneous coronary intervention
STEMI: ST-segmental elevation myocardial infarction
TIMI: Thrombolysis in myocardial infarction
